# Geographic coverage of male circumcision in western Kenya

**DOI:** 10.1097/MD.0000000000005885

**Published:** 2017-01-13

**Authors:** Adam Akullian, Mathews Onyango, Daniel Klein, Jacob Odhiambo, Anna Bershteyn

**Affiliations:** a Institute for Disease Modeling, Bellevue, WA; b National AIDS and STI Control Program (NASCOP), Nairobi, Kenya.

**Keywords:** HIV prevention, implementation science, spatial analysis, Voluntary Medical Male Circumcision (VMMC)

## Abstract

Voluntary Medical Male Circumcision (VMMC) for human immunodeficiency virus (HIV) prevention has scaled up rapidly among young men in western Kenya since 2008. Whether the program has successfully reached uncircumcised men evenly across the region is largely unknown. Using data from two cluster randomized surveys from the 2008 and 2014 Kenyan Demographic Health Survey (KDHS), we mapped the continuous spatial distribution of circumcised men by age group across former Nyanza Province to identify geographic areas where local circumcision prevalence is lower than the overall, regional prevalence. The prevalence of self-reported circumcision among men 15 to 49 across six counties in former Nyanza Province increased from 45.6% (95% CI = 33.2–58.0%) in 2008 to 71.4% (95% CI = 67.4–75.0%) in 2014, with the greatest increase in men 15 to 24 years of age, from 40.4% (95% CI = 27.7–55.0%) in 2008 to 81.6% (95% CI = 77.2–85.0%) in 2014. Despite the dramatic scale-up of VMMC in western Kenya, circumcision coverage in parts of Kisumu, Siaya, and Homa Bay counties was lower than expected (*P* < 0.05), with up to 50% of men aged 15 to 24 still uncircumcised by 2014 in some areas. The VMMC program has proven successful in reaching a large population of uncircumcised men in western Kenya, but as of 2014, pockets of low circumcision coverage still existed. Closing regional gaps in VMMC prevalence to reach 80% coverage may require targeting specific areas where VMMC prevalence is lower than expected.

## 1 Introduction

Voluntary Medical Male Circumcision (VMMC) reduces the risk of HIV acquisition in men by ∼60%.^[1–3]^ Many countries in sub-Saharan Africa have begun a rapid scale-up VMMC in high HIV prevalence areas where circumcision coverage is low.^[4]^ Though a majority of the Kenyan population traditionally practices male circumcision, the burden of HIV is highest in regions where circumcision is not traditionally practiced. The Kenyan national VMMC program has set a target of 80% prevalence of VMMC among men ages 15 to 49 in high HIV prevalence areas.^[5]^ Between 2008 and 2013, 793,000 VMMCs were conducted in Kenya,^[6]^ increasing the proportion of men who reported being circumcised nationwide from 85% in 2007 to 92.6% in 2014.^[7,8]^

The Nyanza region in western Kenya has the highest adult HIV prevalence in Kenya (15.1% among adults ages 15–49 in 2012),^[9]^ and approximately half of men in the age group 15 to 49 years were uncircumcised prior to the start of the national VMMC program.^[10]^ Since the start of the national VMMC program in 2008, the prevalence of circumcised men ages 15 to 49 years in Nyanza increased from 45% to 72% in 2014,^[8]^ with the largest absolute increase (25.2%) among men aged 15 to 24 years.^[7]^ Over the same time period, VMMC services initially only available at fixed provincial, district, or sub-district hospitals were expanded through a mobile program to reach schools and community centers.^[11]^

Despite the large scale-up of VMMC in western Kenya, limited data exist on whether the program has reached uncircumcised men evenly across former Nyanza Province. Using data from two Kenyan Demographic Health Surveys (KDHS), we mapped the continuous spatial distribution of circumcision prevalence among men residing in former Nyanza Province, Kenya, and tested the hypothesis that VMMC prevalence is not homogeneously distributed across the region. Our results can inform the focused allocation of VMMC resources where they are most needed in order to achieve the greatest public health benefit.^[12]^

## 2 Methods

Data from two Kenya Demographic and Health Surveys (KDHS)^[13]^ from 2008 and 2014 were used for this cross-sectional analysis. This analysis was exempt from Institutional review board approval as it relied on de-identified data. Details on the two-stage cluster sampling designed are explained elsewhere.^[14]^ Self-reported circumcision status was the primary outcome of interest. The prevalence of self-reported circumcision was estimated across key demographic groups and indicators of sexual risk behavior including recent sexual activity (never had sex, active in last four weeks, and not active in last four weeks); age at first sex; number of sexual partners in last 12 months (none, 1, 2+); ever tested for HIV; paid for sex in the last 12 months; and concurrent sex partners in the last 12 months (defined by the DHS as “the proportion of men with more than one ongoing sexual partnership at the point in time six months before the interview”^[15]^). Additionally, demographic and sexual risk indicators were compared between those who self-reported having been circumcised (regardless of method) and those who were uncircumcised at time of interview for men surveyed during the 2014 KDHS and who resided in four counties (Siaya, Homa Bay, Migori, and Kisumu) targeted for VMMC scale-up.

Spatial modeling of circumcision prevalence included all men aged 15 to 49 years surveyed in either 2008 or 2014 who resided in six counties of former Nyanza province: Kisumu, Siaya, Homa Bay, Migori, Kisii, and Nyamira. Kisii, Nyamira, and parts of Migori are predominantly comprised of Kisii and Kuria ethnic groups, who practice traditional circumcision. Prevalence of circumcision in those counties is expected to be close to 100%. The residence of each survey respondent was geo-located to the latitude and longitude of their corresponding enumeration area (EA), a geographic area that delineates groups of households for counting during the population census. To ensure anonymity and confidentiality of the survey respondents, each EA is randomly displaced up to 2 km in urban areas and up to 5 km in rural areas, with a randomly selected 1% of rural clusters displaced up to 10 km.^[16]^ The outcome of interest for the mapping exercise was self-reported circumcision status at the time of survey.

Smoothed maps of the fitted prevalence of self-reported circumcised men in two age categories (15–24 and 25–49) were generated separately for the years 2008 and 2014 using a locally weighted regression smoother in a generalized additive model (GAM) framework for binary outcome data^[17]^ and a random effect for the enumeration area (cluster-level variable). Fitted prevalence estimates were plotted on a grid of former Nyanza Province of ∼5 km resolution and credible intervals were generated by iterating the model under spatial randomness and plotting the 1-sided, lower 97.5% credible bound to delineate the geographic boundary of regions with significantly lower prevalence of MC compared to the overall prevalence. Inference on the fitted prevalence map is subject to multiple testing, and we thus used a lower alpha level (0.025) to compensate for the inflated type one error rate.^[18]^ Samples sizes were weighted by DHS survey weights and all analyses accounted for the survey design using the R (version 3.2.2) statistical packages: survey^[19]^ and mgcv.^[20]^

Spatial GAMs are commonly used to model geographic patterns of disease burden and risk, considering a variety of spatial sampling schemes, as well as adjustment for spatial confounding variables.^[21–23]^ We did not include additional co-variates in the GAM because we were interested in understanding spatial differences in circumcision coverage regardless of the cause, including those factors related to known spatial determinants of circumcision access and uptake.

## 3 Results

A total of 484 survey respondents (weighted N = 502.6) were included in the 2008 sample and 1649 survey respondents (weighted N = 1501.2) were included in the 2014 sample. The prevalence of self-reported circumcision increased from 45.6% (95% CI: 33.2–58.0%) in 2008 to 71.4% (67.4–75.0%) by 2014 (Table 1). The increase in circumcision prevalence between 2008 and 2014 was primarily in younger men, 15–19: (37.0–81.3%), 20–24: (46.5–82.2%), and 25–29: (39.0–70.9%) from four traditionally noncircumcising counties of former Nyanza Province targeted during the VMMC campaign. Circumcision prevalence increased from 16.0% to 54.7% in Siaya, 33.3% to 58.5% in Kisumu, 25.4% to 71.6% in Migori, and 11.8% to 52.6% in Homa Bay, and the increases were primarily among those who self-reported being of Luo ethnicity (12.9% to 52.8%) (Table 1). Kisii and Nyamira counties maintained high circumcision prevalence at or close to 100%. Little to no change in prevalence was observed in men over 34 years of age. Circumcision prevalence, initially greater in urban areas compared to rural areas in 2008 (56.5% vs 44.2%), increased to upwards of 70% in both areas by 2014.

**Table 1:**
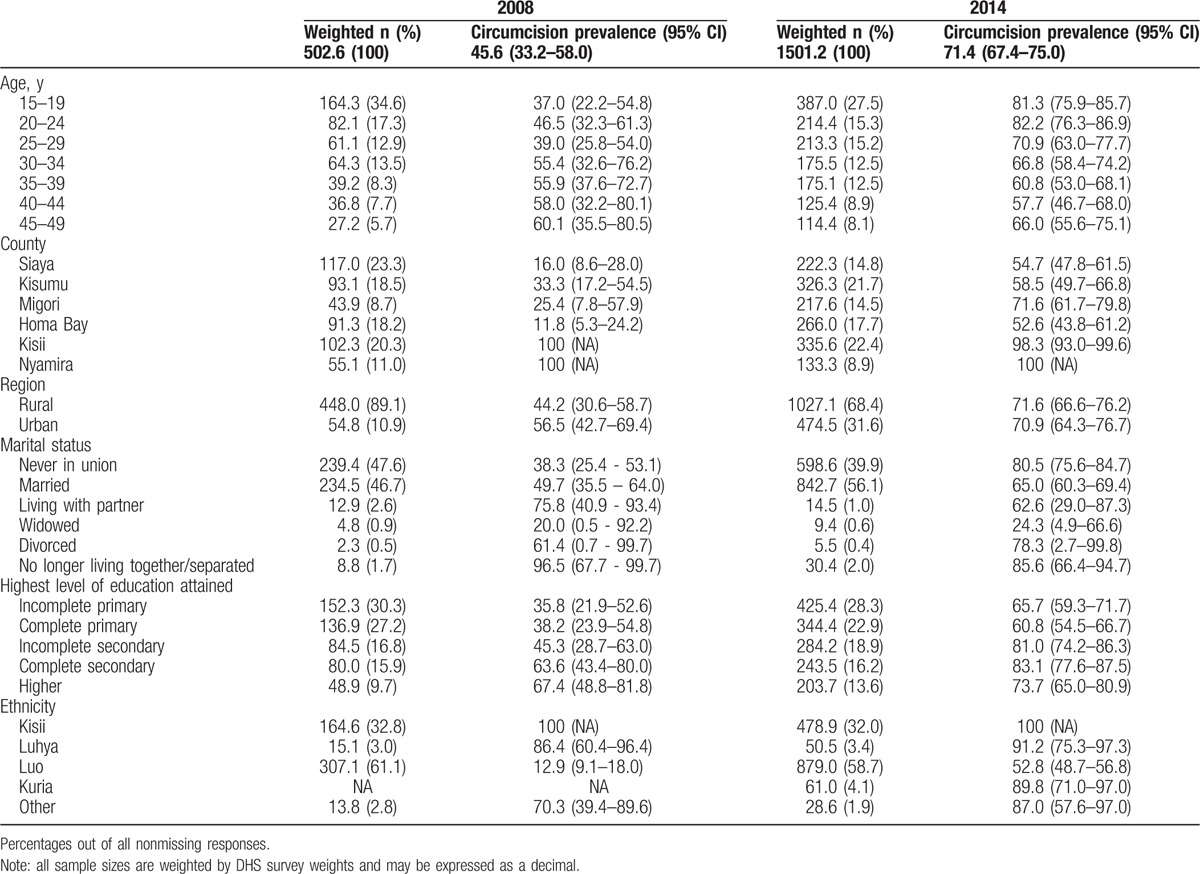
Prevalence of self-reported male circumcision in 2008 and 2014 DHS surveys by key demographic groups.

The distribution of key sexual risk-behaviors among men residing in Siaya, Homa Bay, Kisumu, and Migori Counties in 2014 is shown in Table 2. Circumcised men aged 15 to 24 years were similar to uncircumcised men of the same age with respect to sexual risk behavior, differing only in that they were more likely (*P* = 0.005) to self-report having ever been tested for HIV (84.6%) compared to uncircumcised men of the same age group (72.6%). Circumcised and uncircumcised men aged 15 to 24 years were similar with respect to age (65% were 15–19 in both groups), county of residence, marital status, and level of education. No differences in self-reported sexual risk behavior were observed between circumcised and uncircumcised men aged 15 to 24 years. Circumcised men aged 25 to 49 years tended to have a higher level of education than uncircumcised men of the same age (20.0% completed secondary education compared to 9.2%, respectively, *P* = 0.023) and were less likely to self-report concurrent sexual partners in the last 12 months (52.2% vs 74.7% respectively, *P* = 0.021). There was no evidence of difference in other sexual risk behaviors or in the prevalence of having ever tested for HIV, which was above 90% in both groups.

**Table 2:**
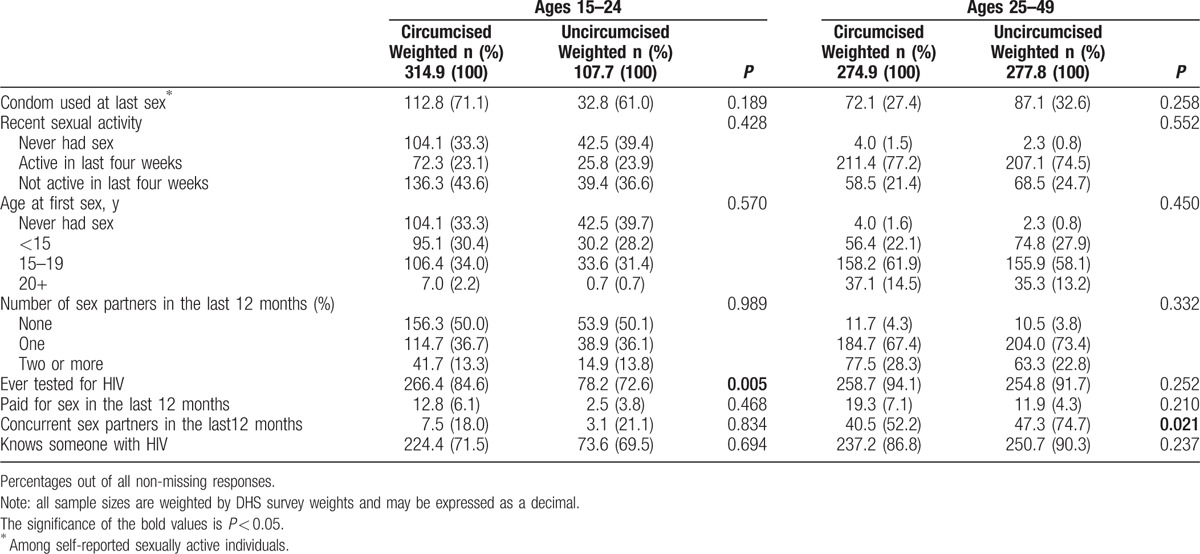
Age-group-specific comparison of sexual risk behaviors between self-reported circumcised and uncircumcised men in four traditionally noncircumcising counties in western Kenya in 2014.

Table 3 shows the distribution of circumcision-specific factors in 2014 across four traditionally noncircumcising counties. Among those aged 15 to 24 years at the time of the survey, 50.4% received circumcision at age 15 to 19 years and 30.6% received circumcision between ages 10 and 14 years. The majority of those aged 15 to 24 years received circumcision after 2007 (84.5%), were circumcised in a health facility (86.9%), and were circumcised by a health worker or professional (87.8%), with fewer circumcised by a traditional practitioner or family friend (11.4%). Among circumcised men aged 25 to 49 years, 32.3% received circumcision after age 25, and more than half were circumcised before age 20. Though the majority of circumcised men aged 25 to 49 years received circumcision before the scale-up of VMMC in 2008, a majority of those circumcisions were reported to have been conducted by health workers (71.1%) and/or in a health facility (70.5%).

**Table 3:**
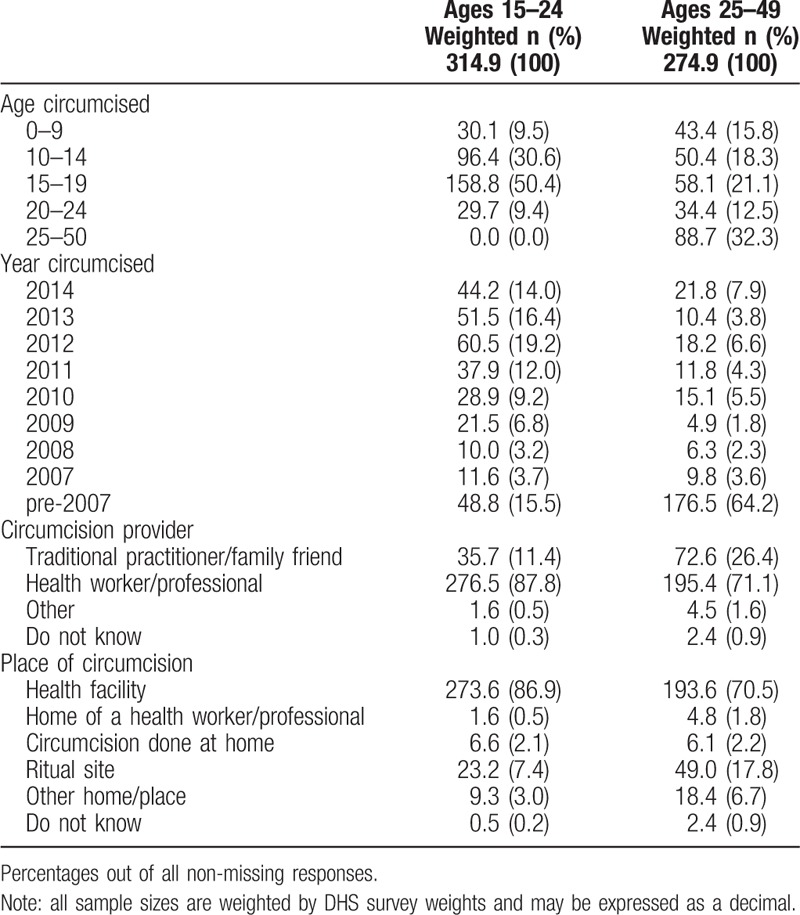
Age-group-specific comparison of circumcised-specific factors of men who self-reported being circumcised in 2014 among four counties in former Nyanza province targeted during the VMMC campaign.

**Figure 1:**
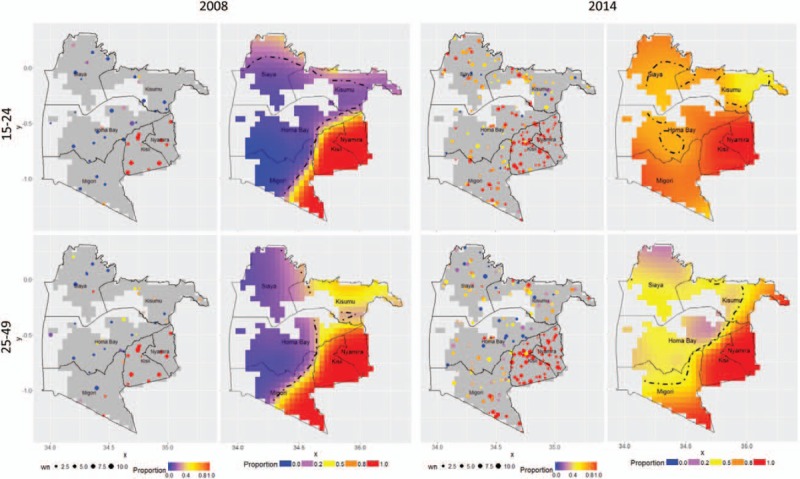
Weighted sample sizes (wn) and smoothed proportions of men age 15 to 24 (top) and 25 to 49 (bottom) who reported to be circumcised prior to Voluntary Medical Male Circumcision (VMMC) scale-up in 2008 (left) and in 2014 (right). Significantly lower than expected departures from the mean prevalence circumcised indicated by one-sided 97.5% credible bound (thick dashed lines). VMMC = Voluntary Medical Male Circumcision.

Figure 1 shows the continuous spatial prevalence of men aged 15 to 24 years and 25 to 49 years who reported to be circumcised in the 2008 and 2014 Kenya Demographic and Health Surveys. In 2008, the lowest prevalence of circumcised men were found in Homa Bay, Kisumu, Migori, and Siaya. These counties experienced the greatest increase in the proportion of circumcised men ages 15 to 24 years, and to a lesser extent, 25 to 49 years. By 2014, the prevalence of circumcised men aged 15 to 24 years was above the 80% MC target across most of the region except for parts of Siaya, Kisumu, and Homa Bay, where the prevalence of circumcised men was significantly lower than the overall mean prevalence (*P* < 0.025). In these regions, 25% to 50% of men aged 15 to 24 years remained uncircumcised in 2014. Among older men, ages 25 to 49 years, 20% to 50% reported being circumcised in 2014 across most of the traditionally noncircumcising regions of Siaya, Homa Bay, western Migori, and western Kisumu.

## 4 Discussion

The VMMC program in Kenya has achieved great gains where it is most needed for HIV prevention. Our mapping results indicated a clear boundary in circumcision status between traditionally circumcising regions of western Kenya, primarily in the counties of Kisii and Nyamira and portions of Migori, and regions with counties with the lowest circumcision prevalence and highest HIV prevalence in Kenya—Homa Bay, Siaya, western Migori, and Kisumu.^[8]^ By 2014, the boundary could no longer be discerned from DHS data for 15 to 24-year-old men, although some regions remained where VMMC coverage was significantly lower than surrounding areas. Among men aged 25 to 49 years, circumcision prevalence in traditionally noncircumcising regions rose, but did not approach the levels of traditionally circumcising regions.

The prevalence of circumcision increased remarkably in men under 30 years of age, more than doubling among men 15 to 19 years of age to reach over 80% in 2014. This reflects the younger target population of the VMMC program.^[5]^ With continued maintenance of high VMMC coverage in young age groups, the prevalence of VMMC in older men will rise over time as young circumcised men age into older groups, thereby providing protection against HIV in the most at-risk male age groups. Circumcised men aged 15 to 24 years were similar to uncircumcised men of the same age across all measured demographic and sexual risk behavior indicators, and differed only with respect having ever tested for HIV – likely a consequence of voluntary testing and counseling being offered to VMMC clients. Our results are consistent with other studies that have found no difference in sexual risk behaviors in circumcised versus uncircumcised men.^[24,25]^

Mapping population-level VMMC coverage over time can show temporal progress and geographic gaps in the VMMC scale-up. The spatial information can also be used to prioritize regions with low-prevalence and high HIV risk, which can further amplify the population-level effect of VMMC programs.^[26]^ Geographic heterogeneities may furthermore indicate regions with limited access to VMMC facilities.^[27]^ Given the geographically heterogeneous nature of the HIV epidemic, the Joint United Nations Programme on HIV/AIDS (UNAIDS) has called for renewed research efforts into the use of spatial analysis in epidemiology and health services research to identify populations both at greatest risk of HIV infection and in greatest need of prevention services.^[28]^ A number of modeling studies have investigated the potential impact of targeting VMMC at key age-groups^[29]^ and geographic regions where MC prevalence is low.^[30,31]^ Our study is among the first to document small-scale geographic gaps in VMMC coverage. Further study can clarify the determinants of geographic differences in circumcision coverage, whether they be differences in availability or demand and uptake of VMMC services.

A limitation of this analysis is that the 2014 DHS did not include HIV testing, and thus we could not restrict the analysis to HIV-negative men, who are the target of VMMC for HIV prevention. In 2012, HIV prevalence in the regions we mapped was very high among men aged 15 to 54 years: 22.3% in Homa Bay, 26.1% in Siaya, 19.7% in Kisumu, and 14.7% in Migori. Although VMMC is not denied to HIV-positive men, the highest-quality evidence of the benefit of VMMC for HIV prevention is among HIV-negative men. Second, though the DHS aims to capture a representative sample by providing sampling weights to correct for under/oversampling, there may still be some degree of underrepresentation of certain groups, namely male economic migrants who are at a high risk of HIV. Furthermore, the circumcision status of men is based on the self-report and not verified by genital exam. Finally, the sample sizes of many enumeration districts was small (<10). The smooth spatial model, however, is advantageous with sparse data in that it borrows information across continuous space, resulting in more stable estimates of prevalence. Still, smoothed estimates need to be interpreted with caution where there are limited data points.^[32]^

Geographic prioritization VMMC services has been a hallmark of the current effort to target high-risk hotspots of HIV transmission in SSA. As VMMC programs continue to expand across target regions, monitoring small-scale regional gaps in the coverage, availability, and uptake of VMMC services will be essential to ensure the most effective and efficient scale-up of VMMC for HIV prevention.

## References

[1] Auvert B Taljaard D Lagarde E Randomized, controlled intervention trial of male circumcision for reduction of HIV infection risk: the ANRS 1265 Trial. PLoS Med 2005;2:e298. 16231970 10.1371/journal.pmed.0020298 PMC1262556

[2] Bailey RC Moses S Parker CB Male circumcision for HIV prevention in young men in Kisumu, Kenya: a randomised controlled trial. Lancet 2007;369:643–56. 17321310 10.1016/S0140-6736(07)60312-2

[3] Gray RH Kigozi G Serwadda D Male circumcision for HIV prevention in men in Rakai, Uganda: a randomised trial. Lancet 2007;369:657–66. 17321311 10.1016/S0140-6736(07)60313-4

[4] WHO. Voluntary medical male circumcision for HIV prevention in 14 priority countries in East and Southern Africa. In; 2015.

[5] Government of Kenya MoH. National AIDS and STI Control Program. National Voluntary Medical Male Circumcision Strategy, 2014/15-2018/19. In. Nairobi, Kenya.

[6] Benson FN Onyango M Stover J Evaluating the Impact of the Voluntary Medical Male Circumcision Program in Kenya. In: *ICASA 2015*. Harare, Zimbabwe; 2015.

[7] Galbraith JS Ochieng A Mwalili S Status of voluntary medical male circumcision in Kenya: findings from 2 nationally representative surveys in Kenya, 2007 and 2012. J Acquir Immune Defic Syndr 2014;66 (Suppl 1):S37–45. 24732820 10.1097/QAI.0000000000000121 PMC4794989

[8] Kenya National Bureau of Statistics MoH, National AIDS Control Council, Kenya Medical Research Institute, National Council for Population and Development, Nairobi, Kenya, and The DHS Program, ICF International, Rockville, Maryland, USA, Kenya DHS, 2014—Final Report, 2015.

[9] Kimanga DO Ogola S Umuro M Prevalence and incidence of HIV infection, trends, and risk factors among persons aged 15–64 years in Kenya: results from a Nationally Representative Study. J Acquir Immune Defic Syndr (1999) 2014;66:S13–26. 10.1097/QAI.0000000000000124 PMC4794992 24445338

[10] Maina WK Kim AA Rutherford GW Kenya AIDS Indicator Surveys 2007 and 2012: implications for public health policies for HIV prevention and treatment. J Acquir Immune Defic Syndr 2014;66 (suppl 1):S130–7. 24732817 10.1097/QAI.0000000000000123 PMC4784700

[11] Centers for Disease Control and Prevention (CDC) Progress in voluntary medical male circumcision service provision—Kenya, 2008–2011. Morb Mortal Wkly Rep 2012;61:957–61. 23190568

[12] Anderson S-J Cherutich P Kilonzo N Maximising the effect of combination HIV prevention through prioritisation of the people and places in greatest need: a modelling study. Lancet 2014;384:249–56. 25042235 10.1016/S0140-6736(14)61053-9

[13] National Statistical Service MoH, and ICF International. Kenya Demographic and Health Survey 2008/2014 [Dataset]. In. Calverton, Maryland: ICF International; 2008 and 2014.

[14] ICF International. Demographic and Health Survey Sampling and Household Listing Manual. In. Calverton, Maryland: MEASURE DHS; 2012.

[15] Fishel JD Ladys Ortiz Bernard Barrère Measuring Concurrent Sexual Partnerships: Experience of the MEASURE DHS Project to Date. DHS Methodological Reports No. 7. In. Calverton, Maryland, USA: ICF International; 2012.

[16] Gething P Andy Tatem Tom Bird Clara R Burgert-Brucker. Creating Spatial Interpolation Surfaces with DHS Data DHS Spatial Analysis Reports No. 11. In. Rockville, Maryland: ICF International; 2015.

[17] Webster T Vieira V Weinberg J Method for mapping population-based case-control studies: an application using generalized additive models. Int J Health Geogr 2006;5:26. 16764727 10.1186/1476-072X-5-26 PMC1526437

[18] Young RL Weinberg J Vieira V Generalized additive models and inflated type I error rates of smoother significance tests. Comput Stat Data Aanal 2011;55:366–74. 10.1016/j.csda.2010.05.004 PMC2952638 20948974

[19] Lumley T Analysis of Complex Survey Samples. In. package version 1.12 ed; 2016.

[20] Wood S Mixed GAM Computation Vehicle with GCV/AIC/REML Smoothness Estimation. In. package version 1.8-16 ed; 2016.

[21] Akullian A Kohler P Kinuthia J Geographic distribution of HIV stigma among women of childbearing age in rural Kenya. AIDS 2014;28:1665–72. 24835356 10.1097/QAD.0000000000000318 PMC4394656

[22] Siqueira-Junior JB Maciel IJ Barcellos C Spatial point analysis based on dengue surveys at household level in central Brazil. BMC Public Health 2008;8:361. 18937868 10.1186/1471-2458-8-361 PMC2576465

[23] Zanin L Radice R Marra G Modelling the impact of women's education on fertility in Malawi. J Population Econ 2015;28:89–111.

[24] Westercamp N Agot K Jaoko W Risk compensation following male circumcision: results from a two-year prospective cohort study of recently circumcised and uncircumcised men in Nyanza Province, Kenya. AIDS Behav 2014;18:1764–75. 25047688 10.1007/s10461-014-0846-4

[25] Gray R Kigozi G Kong X The effectiveness of male circumcision for HIV prevention and effects on risk behaviors in a posttrial follow-up study. AIDS 2012;26:609–15. 22210632 10.1097/QAD.0b013e3283504a3f PMC4296667

[26] Hallett TB Singh K Smith JA Understanding the impact of male circumcision interventions on the spread of HIV in southern Africa. PLoS One 2008;3:e2212. 18493593 10.1371/journal.pone.0002212 PMC2387228

[27] Golub G Herman-Roloff A Hoffman S The relationship between distance and post-operative visit attendance following medical male circumcision in Nyanza province, Kenya. AIDS Behav 2015;20:2529–37. 10.1007/s10461-015-1210-z PMC5590645 26424709

[28] UNAIDS Reference Group on Estimates MaP. Identifying Populations at Greatest Risk of Infection— Geographic Hotspots and Key Populations In. Geneva, Switzerland; 2013.

[29] Kripke K Okello V Maziya V Voluntary medical male circumcision for HIV prevention in Swaziland: modeling the impact of age targeting. PLoS One 2016;11:e0156776. 27410687 10.1371/journal.pone.0156776 PMC4943626

[30] Kripke K Chen P-A Vazzano A Cost and impact of voluntary medical male circumcision in South Africa: focusing the program on specific age groups and provinces. PLoS One 2016;11:e0157071. 27409079 10.1371/journal.pone.0157071 PMC4943592

[31] Kripke K Vazzano A Kirungi W Modeling the impact of Uganda's safe male circumcision program: implications for age and regional targeting. PLoS One 2016;11:e0158693. 27410234 10.1371/journal.pone.0158693 PMC4943628

[32] Larmarange J Bendaud V HIV estimates at second subnational level from national population-based surveys. AIDS (London, England) 2014;28:S469–76. 10.1097/QAD.0000000000000480 PMC4247267 25406750

